# PD‐L1‐Inhibitor‐Associated Hidradenitis Suppurativa

**DOI:** 10.1111/cup.70068

**Published:** 2026-02-05

**Authors:** Annie Jin, Katherine T. Steele, Emily Y. Chu

**Affiliations:** ^1^ Department of Dermatology Hospital of the University of Pennsylvania Philadelphia Pennsylvania USA

**Keywords:** cutaneous adverse reaction, hidradenitis suppurativa, immune checkpoint inhibitor, PD‐L1

Immune checkpoint inhibitors (ICIs) are increasingly being utilized to treat a variety of advanced malignancies, including but not limited to melanoma, small cell and non‐small cell lung cancer, renal cell carcinoma, and urothelial carcinomas. Many immune‐related cutaneous adverse events (irCAEs) are associated with Programmed cell death protein 1 (PD‐1) and Programmed death‐ligand 1 (PD‐L1) inhibitors, including morbilliform eruptions, pruritus, lichenoid dermatitis, psoriasiform dermatitis, bullous pemphigoid, sarcoidal granulomatous reactions, lupus erythematosus and dermatomyositis‐like reactions, vitiligo‐like skin hypopigmentation/depigmentation, and rarely Sweet syndrome [1, 2, 3]. The PD‐1 inhibitor nivolumab has rarely been associated with new onset hidradenitis suppurativa (HS) [4, 5]. Herein, we report a case of a 73‐year‐old female patient who developed hidradenitis suppurativa approximately 3 months after initiation of treatment with atezolizumab, a PD‐L1 inhibitor.

A 73‐year‐old female patient with stage IVB small‐cell lung cancer presented to the dermatology clinic for evaluation of nodules in the axillae and groin. Her medical history was notable for concomitant obesity and Graves' disease, and a > 50 pack‐year smoking history. After diagnosis of metastatic lung cancer with soft tissue and brain involvement, the patient completed 4 cycles of carboplatin, etoposide, and atezolizumab over a 2‐month period, then started maintenance treatment with atezolizumab 1200 mg every 3 weeks. She first noticed the appearance of erythematous nodules in her right axilla and right inguinal fold 16 days after her last dose of combined carboplatin, etoposide, and atezolizumab therapy, and subsequently developed similar red nodules in the contralateral axilla and inguinal regions 1 week following a dose of atezolizumab alone (Figure 1A,B). A prednisone taper was initiated by her oncology team prior to dermatology evaluation. Two punch biopsy specimens were obtained from nodules in her left axilla for histopathologic evaluation and tissue culture to rule out an infectious process. The biopsy specimen showed a perivascular, peri‐apocrine, and interstitial inflammatory infiltrate composed of lymphocytes, plasma cells, and neutrophils in the superficial to deep dermis (Figure 2A,B). Grocott's methenamine silver (GMS) and Fite stains were negative for organisms. Tissue culture did not show organism growth. A diagnosis of PD‐L1‐inhibitor‐associated hidradenitis suppurativa was made. The cutaneous lesions improved following temporary cessation of atezolizumab and treatment with doxycycline. She continued doxycycline therapy and also received a prednisone taper prior to restarting the PD‐L1 inhibitor therapy several weeks later and did not develop additional cutaneous lesions.

**FIGURE 1:**
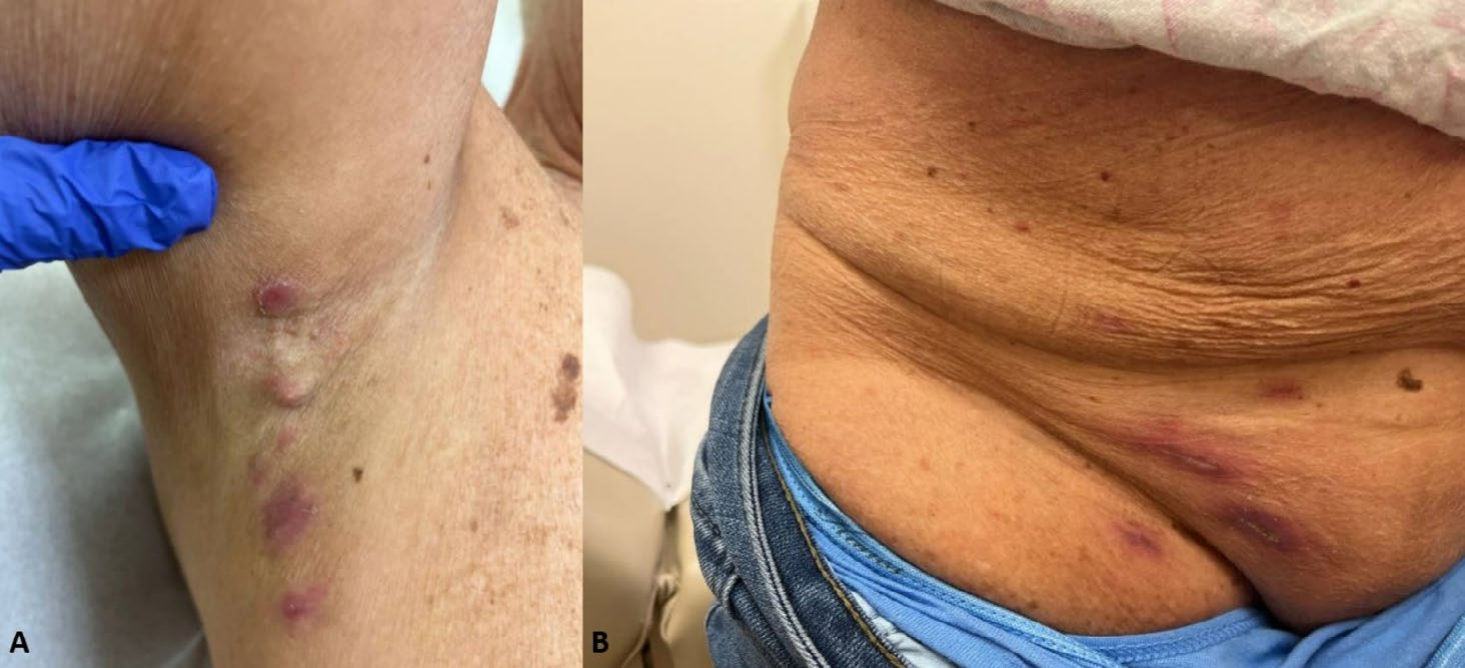
Erythematous nodules in the axillae and inguinal folds. (A and B, respectively).

**FIGURE 2:**
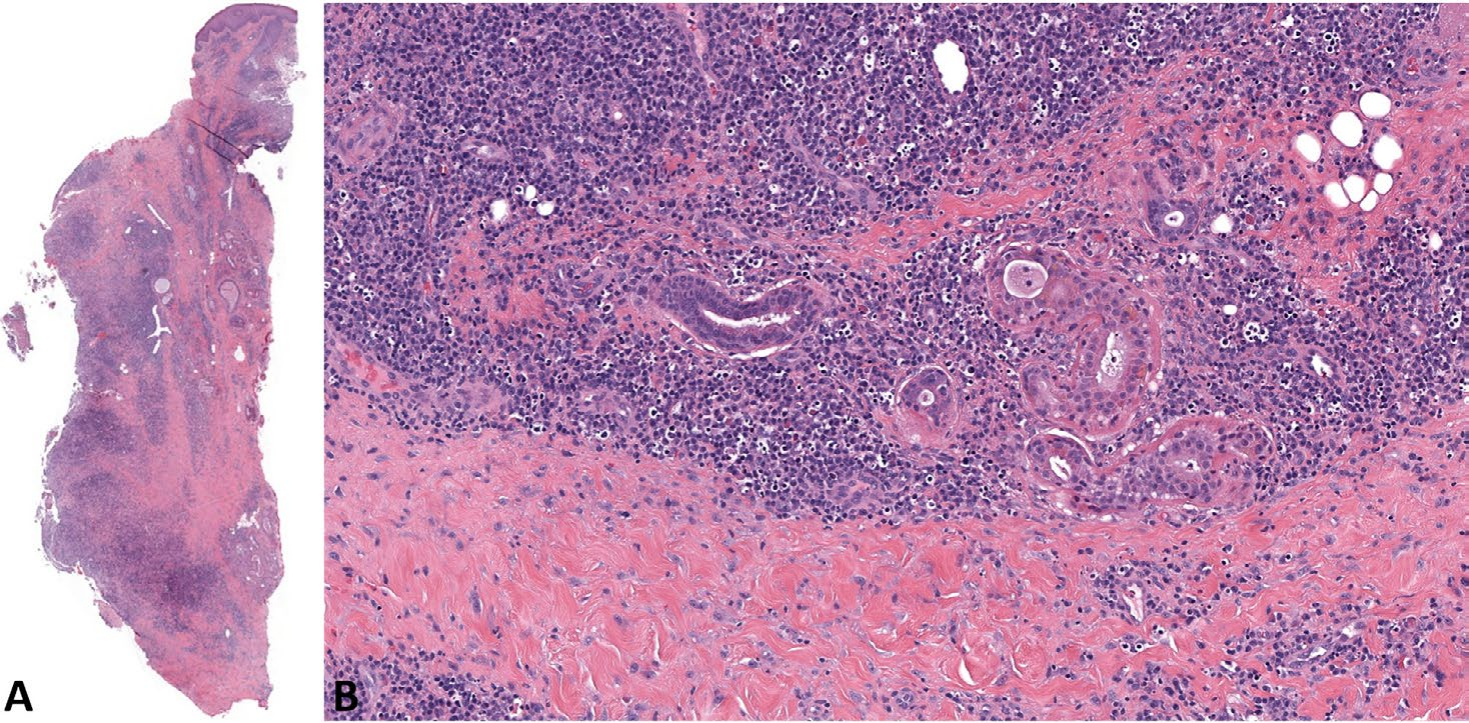
(A) Perivascular and periadnexal infiltrate with associated fibrosis (hematoxylin and eosin stain, original magnification ×20). (B) Perivascular and periadnexal mixed inflammatory infiltrate composed of neutrophils, plasma cells, and lymphocytes with associated fibrosis (hematoxylin and eosin stain, original magnification ×100).

The histopathologic features of HS are variable, especially depending on the stage and extent of disease, and not particularly well characterized since the diagnosis is often established clinically [6]. Histopathology may reveal follicular hyperkeratosis and perifollicular inflammation, particularly in earlier lesions. Suppurative inflammation in association with sinus tracts and cystic structures, mixed dermal inflammation, granulation tissue, and fibrosis may all be observed in later lesions. Our patient's biopsy findings of a dense mixed infiltrate of lymphocytes, plasma cells, and neutrophils combined with the clinical presentation supported the diagnosis of HS.

Immune checkpoint inhibitors (ICIs), including PD‐L1 inhibitors, are associated with a broad spectrum of autoimmune and other immune‐mediated cutaneous reactions, including conditions which affect adnexal structures including alopecia areata and hypohidrosis/anhidrosis [7]. While our patient was treated with carboplatin and etoposide in addition atezolizumab, the PD‐LI inhibitor was thought to be the most likely culprit in the development HS based on the rare published reports of nivolumab‐associated hidradenitis suppurativa. This was further supported by the improvement of the cutaneous lesions following atezolizumab cessation and doxycycline therapy. Our patient, similar to the two previously reported cases of nivolumab‐associated HS, did not have a history of HS prior to beginning the medication, although she had risk factors associated with HS including obesity and a significant smoking history. The latency period between PD‐L1 inhibitor initiation and onset of HS lesions in our case was similar to those of previously reported cases of PD‐1‐inhibitor‐induced HS—1–3 months [4, 5]. ICI‐associated cutaneous adverse reactions may present shortly after initiation of therapy, but have also been well documented to occur in a delayed fashion months or even years after beginning treatment, and in some cases after immunotherapy has been discontinued [8].

IL‐17 is a key cytokine in HS pathogenesis, with lesional and perilesional HS skin demonstrating increased Th17 cells [9]. The Th17 pathway has been implicated in the pathogenesis of ICI‐induced colitis and PD‐1‐inhibitor‐induced psoriasis [1, 10]. Thus, the Th17 pathway has been implicated in the pathogenesis of PD‐1‐inhibitor‐induced HS. Given the similarity in mechanism between PD‐1 and PD‐L1 inhibitors, alteration of the Th17 pathway may account for development of HS in our PD‐L1‐inhibitor‐treated patient as well. With increasing use of immunotherapies in the treatment of advanced malignancies, it is important for dermatopathologists to be aware of the spectrum of irCAEs that may arise for accurate diagnosis and subsequent clinical management.

## Funding

The authors have nothing to report.

## Ethics Statement

The authors have nothing to report.

## Conflicts of Interest

The authors declare no conflicts of interest.

## Data Availability

Data sharing not applicable to this article as no datasets were generated or analyzed during the current study.
